# Alveolar Paratesticular Rhabdomyosarcoma in an Adult Patient Mimicking Epididymo-Orchitis: A Case Report and a Literature Review

**DOI:** 10.7759/cureus.24786

**Published:** 2022-05-06

**Authors:** Ahmed S Al Ghamdi, Naif M Alharbi, Khalil F Miyajan, Aseel A Hazzazi, Ali A Fadel, Numan Tabba

**Affiliations:** 1 Urology, Royal Commission Yanbu Medical Centre, Yanbu, SAU; 2 Family Medicine, Ministry of Health, Makkah, SAU; 3 Faculty of Medicine, Umm Al Qura University, Makkah, SAU; 4 Urology, International Medical Center, Jeddah, SAU

**Keywords:** epididymo-orchitis, adult onset, paratesticular mass, radical orchiectomy, alveolar rhabdomyosarcoma

## Abstract

The majority of patients with paratesticular rhabdomyosarcoma (RMS) present in the pediatric age group with a unilateral, painless, palpable scrotum mass. By contrast, cases of RMS presenting as painful edema are rare. We present a case of alveolar paratesticular RMS in a 30-year-old man who had been suffering from a painful swelling of the scrotum on the left side for two years and a preceding mass four months before visiting the clinic. Complete resection of the left epididymal mass was performed through a left inguinal incision. The histopathological and immunohistochemical examination of the mass revealed alveolar RMS of the paratesticular region. Urologists should be aware that paratesticular RMS may present in adults with atypical symptoms such as scrotal pain and edema, especially in those who do not respond to antibiotics. Hence, such patients should have an additional evaluation.

## Introduction

Rhabdomyosarcoma (RMS) is a primitive pediatric malignant soft tissue sarcoma of the skeletal muscle phenotype that originates from a primitive mesenchymal cell. Most cases are diagnosed at pediatric age, and it is rare in adults [1,2]. RMS accounts for approximately 6.5% of malignancies in patients under the age of 15 and 0.03% of solid tumors in adults [3,4]. Paratesticular RMS develops from the epididymis, testicular envelopes, and spermatic cord and accounts for only 7-10% of genitourinary RMS tumors [5]. It usually manifests as a painless, rapidly growing mass in the scrotum or inguinal canal. However, to the best of our knowledge, only a few previous cases have involved painful symptoms [6-8]. Here, we present the case of a 30-year-old Saudi male with painful paratesticular RMS that comes with an unusual presentation in an atypical age group.

## Case presentation

A 30-year-old male patient presented with intermittent mild pain in the left scrotum for two years; approximately four months before presentation, he felt a non-painful mass that gradually increased in size. He was started on antibiotics (ciprofloxacin for four weeks and doxycycline for two weeks) for possible epididymo-orchitis. However, the patient did not experience any improvement in scrotal swelling and requested a scrotal ultrasound, which revealed an epididymal mass. The patient was then presented to the urology unit of the Royal Commission Medical Center Yanbu’s Department of Surgery for additional evaluation.

On physical examination, the left hemiscrotum appeared normal skin color and had no signs of acute inflammation. A palpable, hard, and movable mass measuring approximately 4×4 cm was located in the left epididymis tail below and was attached to the left testis but not to the scrotal skin.

**Figure 1:**
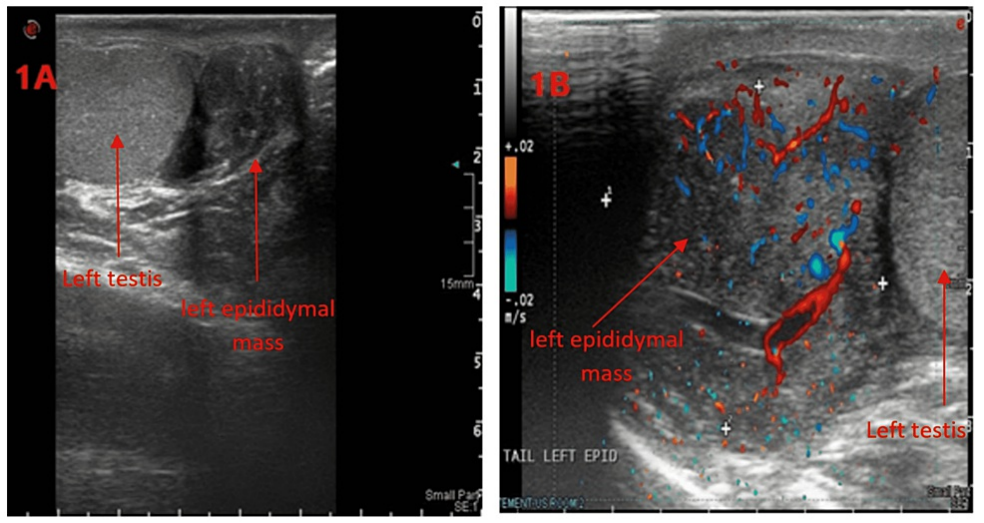
Testicular ultrasound (A) Ultrasound of the scrotum revealed left testis with multiple tiny parenchymal calcifications and left epididymal tail bulky mass contained multiple calcific foci as well. (B) The tail and body of the left epididymis were diffusely enlarged with hypoechoic echotexture of size (3.5×2.8 cm) with significantly increased vascularity with focal hypoechoic lesions with distinct margins, mostly consistent with inflammation.

**Figure 2:**
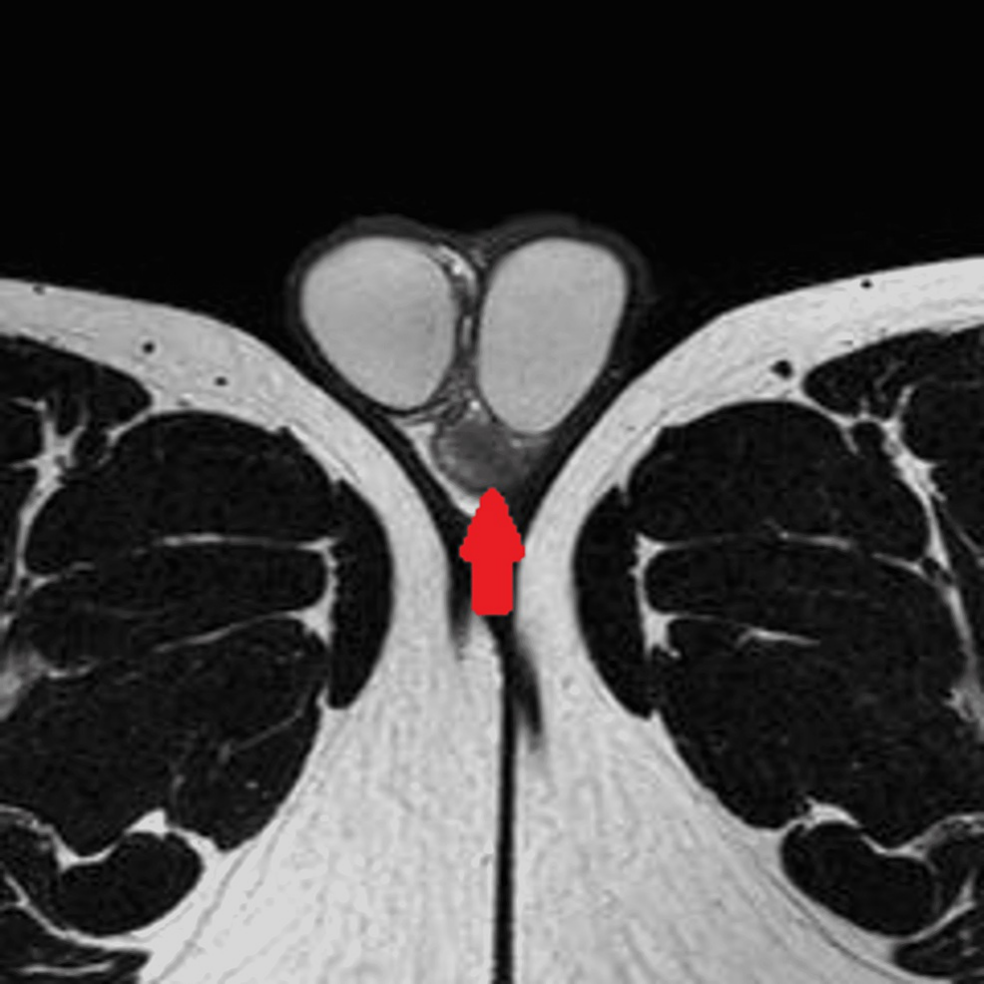
Scrotal MRI Scrotal MRI revealed a 3×1.9 cm extratesticular mass in the tail of the left epididymis. The mass demonstrates dark signal intensity on T1- and T2-weighted sequences. It is separable from the testicle and not attached to the scrotal wall. MRI, magnetic resonance imaging.

**Figure 3:**
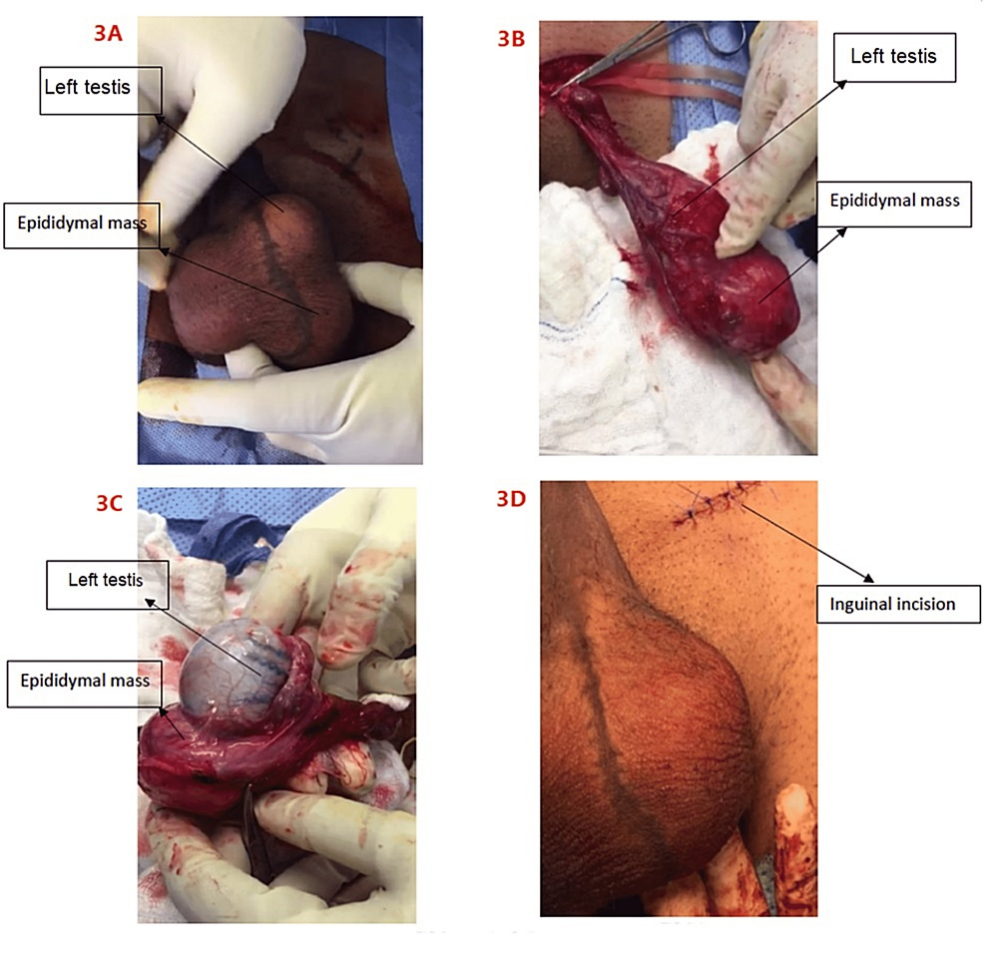
Complete resection of the left epididymal mass (A) Scrotum with left testis above the left epididymal mass, which was a hard mass. (B) Through the left inguinal incision and after clamping the cord, the left tunica vaginalis component was extracted smoothly. (C) An opening of the tunica vaginalis revealed a hydrocele with a hard mass measuring 4×4 cm and originating from the left epididymal tail. (D) Closure of the left inguinal incision was done after left epididymal tail mass resection.

Laboratory tests revealed that beta-human chorionic gonadotropin (ß-HCG), lactate dehydrogenase (LDH), and alfa-fetoprotein (AFP) were in the normal range. The semen concentration was 5 ml, 40 million cells/ml, active 60%, and non-motile 20%, otherwise unremarkable. On imaging, the testicular ultrasound revealed a left testis with numerous tiny parenchymal calcifications with normal vascularity. Further, it showed a bulky mass in the left epididymal tail with numerous calcific foci with significantly increased vascularity, mostly consistent with inflammation (Figure 1). On magnetic resonance imaging (MRI), a 3×1.9 cm extratesticular left epididymal tail mass was discovered without lymphadenopathy (Figure 2) with no metastasis on the chest and abdominal computed tomography (CT). Due to the presence of this left paratesticular mass, the complete resection of the left epididymal mass was performed through a left inguinal incision (Figure 3).

Microscopic examination revealed an unencapsulated, poorly circumscribed tumor formed by cell proliferation. The fibrous stroma had foci of abundant smooth muscle and elastic fibers, which had a desmoplastic appearance and that were infiltrated by inflammatory cells with prominent cytoplasm vacuolation. The first impression was an adenomatoid tumor of the epididymis and free margins from malignancy. As our hospital’s policy requires us to obtain a second opinion to confirm a diagnosis, a specimen was sent to an advanced oncology center. There, immunohistochemistry revealed that the tumor cells were positive for desmin, MyoD1, and myogenin. Cytogenetic testing fluorescence in situ hybridization (FISH) for the FKHR (FOXO1) mutation was positive, confirming the diagnosis of alveolar RMS. Hence, the patient was referred to a more advanced oncology center to complete his treatment.

## Discussion

RMS is a type of malignant soft tissue tumor that develops from striated muscle cells or mesenchymal cells that have been differentiated from striated muscle cells. It is the most common soft tissue sarcoma in children, accounting for approximately 6.5% of malignancies under the age of 15; however, it is extremely rare in adults [4]. RMS types include embryonal (60%), alveolar (20%), pleomorphic (10%), and spindle/sclerosing (10%) [1]. Paratesticular RMS is relatively rare and represents around 7% of genitourinary RMS [9]. Our patient was diagnosed with alveolar paratesticular RMS, which is rarely present in his age group.

Paratesticular RMS typically manifests as a painless epididymal mass or as nonspecific symptoms such as decreased appetite, fatigue, inguinal lymphadenopathy, and weight loss. Pain can occur when paratesticular RMS compresses a nerve. However, pain is extremely rare, occurring in only 7% of patients [10,11]. When paratesticular RMS is associated with painful unilateral scrotal swelling, a misdiagnosis of epididymitis or epididymo-orchitis may occur, as in Lei’s [8] report in which a 19-year-old presented with the painful swelling of the left scrotum for several days that was initially misdiagnosed as epididymitis. As in our case, the patient initially presented with intermittent mild pain in the left scrotum and a nonpainful mass that gradually increased in size, which was misdiagnosed as epidydmo-orchitis. However, antibiotic treatment can decrease scrotal pain, as reported by Lei [8], who found that after two months of antibiotic treatment, the pain improved but the edema did not go away. By contrast, our patient’s condition did not improve, raising concerns about paratesticular RMS.

It is not straightforward to clinically establish a diagnosis of RMS of the testis, epididymis, spermatic cord, or anywhere within the scrotum because it can mimic the symptoms of epididymitis or epididymo-orchitis, which can delay diagnosis [9]. Ultrasound scans are the initial imaging modality for evaluating masses in the testis or paratesticular region. Such scans tend to show increased vascularity or a mass compressing the testis but can lead to confusion with adenomatoid tumors, leiomyoma, and epididymitis [12,13].

Wood and Dewbury [14] described the first ultrasonography findings associated with paratesticular RMS in a 17-year-old male patient. They found increased vascularity in an epididymal mass that they initially misdiagnosed as epididymitis. Then, after six months of progressive scrotal edema, paratesticular RMS was diagnosed. Mak et al. [12] reported that a 14-year-old male patient was misdiagnosed with epididymitis because of their inability to differentiate paratesticular RMS from epididymitis using ultrasound. Additionally, according to Lei [8], a 19-year-old male patient was misdiagnosed with epididymitis because the ultrasound provided insufficient information to make a definitive diagnosis. This demonstrates the difficulty in distinguishing RMS from epididymitis by ultrasound alone. In our case, the patient delayed the diagnosis of RMS by relying solely on an ultrasound carried out by a private clinic. Therefore, more advanced evaluation is critical for patients presenting with epididymitis symptoms or complications such as epididymo-orchitis, especially when they do not respond well to antibiotic therapy.

MRI scans can show a heterogeneously enhancing mass within the testis or a mass in the paratesticular region compressing the testis [13]. Radiology imaging tends to be confirmed by histopathology examinations and immunohistochemistry studies. Routine hematology and biochemistry test results tend to be normal, as do the serum ß-HCG, AFP, and LDH levels. Immunohistochemistry studies may show positive staining for desmin, myoglobin, myosin, MSA, MyoD1, myogenin, and, rarely, cytokeratin [15]. Although histology is still the gold standard for diagnosis and classification, there has been a renewed emphasis on gene translocation to support risk stratification. The fusion of the PAX7 or PAX3 genes with the FOXO1 gene is detected in the majority of alveolar RMS and this predicts a poor prognosis. However, this gene fusion is absent in around 20% of alveolar subtypes [16]. In our patient, the serum ß-HCG, LDH, and AFP levels were normal, and immunostaining revealed positive results for desmin, MyoD1, and myogenin in the tumor cells. Cytogenetic testing (FISH) for the FKHR (FOXO1) mutation confirmed the diagnosis of alveolar RMS.

The treatment of paratesticular RMS includes multimodal therapy with systemic chemotherapy and surgery, radiation therapy, or both to maximize tumor control. Surgical intervention alone produces a 50% two-year relapse-free survival rate before effective chemotherapy [17]. Patients with suspected paratesticular RMS undergo radical orchiectomy via an inguinal incision with the high dissection and ligation of the spermatic cord. The scrotal approach is inadequate due to the risk of microscopic residual disease contamination but is used in up to 25% of cases [18]. The scrotal approach is inadequate due to the risk of microscopic residual disease contamination, but it is used in up to 25% of cases [18]. Following a trans-scrotal approach, primary re-excision, which includes the wide local excision of scar tissue, has traditionally been recommended; however, this frequently results in hemiscrotectomy and the high ligation of the spermatic cord [19,20]. The use of retroperitoneal lymph node dissection (RPLND) in paratesticular RMS is debatable and has evolved over the past 20 years. Approximately 25% of patients with paratesticular RMS are found to have retroperitoneal lymph node disease at presentation. Only those patients with lymphadenopathy on CT underwent RPLND [9].

All patients with paratesticular RMS should be considered for adjuvant chemotherapy treatment. Available protocols include doxorubicin and ifosfamide as well as etoposide, vincristine, actinomycin-D, cyclophosphamide, and cisplatin. According to the European Society for Medical Oncology and Minimal Clinical Recommendations, doxorubicin is the first-line chemotherapy for soft tissue sarcoma, and there is no clear evidence that polychemotherapy is more successful than doxorubicin alone in terms of overall survival. However, a combination of doxorubicin and ifosfamide results in a higher response rate [21,22], reaching up to 86% in recent investigations of extremity-localized RMS in adult patients [23]. Local radiotherapy is recommended in addition to systemic treatment in all patients with histopathologically positive lymph nodes and microscopic residual disease [24].

Regarding our patient, he had a complete resection of the left epididymal mass through a left inguinal incision in our hospital, and he was then referred to an advanced oncology center to complete his treatment when the ultimate diagnosis of left alveolar paratesticular RMS was confirmed. In terms of outcome, the localized disease has a better prognosis than metastases. A poor prognosis is frequently associated with age, particularly in patients aged under one year or over 10 years [15].

## Conclusions

In conclusion, this case highlights an important point: urologists should be aware that paratesticular RMS can present in adults with atypical manifestations that include scrotal pain and edema. Due to the highly aggressive character of paratesticular RMS, additional evaluations are necessary for patients with epidydmo-orchitis symptoms, particularly those who do not respond to antibiotic therapy.
